# Smartphone keyboard dynamics predict affect in suicidal ideation

**DOI:** 10.1038/s41746-024-01048-1

**Published:** 2024-03-01

**Authors:** Loran Knol, Anisha Nagpal, Imogen E. Leaning, Elena Idda, Faraz Hussain, Emma Ning, Tory A. Eisenlohr-Moul, Christian F. Beckmann, Andre F. Marquand, Alex Leow

**Affiliations:** 1 Donders Centre for Cognitive Neuroimaging, Donders Institute for Brain, Cognition and Behaviour, Radboud University, Nijmegen, the Netherlands; 2 Department of Cognitive Neuroscience, Radboud University Medical Centre, Nijmegen, the Netherlands; 3 Department of Psychiatry, University of Illinois at Chicago, Chicago, IL USA; 4 Department of Electronics, Information and Bioengineering, Politecnico di Milano, Milan, Italy; 5 Department of Biomedical Engineering, University of Illinois at Chicago, Chicago, IL USA; 6 Department of Psychology, University of Illinois at Chicago, Chicago, IL USA; 7 Oxford Centre for Functional Magnetic Resonance Imaging of the Brain (FMRIB), University of Oxford, Oxford, UK; 8 Department of Computer Science, University of Illinois at Chicago, Chicago, IL USA

**Keywords:** Diagnostic markers, Data integration

## Abstract

While digital phenotyping provides opportunities for unobtrusive, real-time mental health assessments, the integration of its modalities is not trivial due to high dimensionalities and discrepancies in sampling frequencies. We provide an integrated pipeline that solves these issues by transforming all modalities to the same time unit, applying temporal independent component analysis (ICA) to high-dimensional modalities, and fusing the modalities with linear mixed-effects models. We applied our approach to integrate high-quality, daily self-report data with BiAffect keyboard dynamics derived from a clinical suicidality sample of mental health outpatients. Applying the ICA to the self-report data (104 participants, 5712 days of data) revealed components related to well-being, anhedonia, and irritability and social dysfunction. Mixed-effects models (55 participants, 1794 days) showed that less phone movement while typing was associated with more anhedonia (β = −0.12, *p* = 0.00030). We consider this method to be widely applicable to dense, longitudinal digital phenotyping data.

## Introduction

Traditionally, mental health assessments are administered by professionals in the clinic and therefore occur infrequently, outside the context of an individual’s daily life. The ubiquity of smartphones presents many opportunities for more frequent mental health assessments outside of the clinic^1^. A popular and direct measure of mental state administered through smartphones are self-report questionnaire-style prompts, like ecological momentary assessment (EMA), which repeatedly sample behaviour and experiences in their natural environment, in real-time^2^. As with any measurements that rely on active user engagement, however, EMA imposes burden on the participant and is therefore prone to attrition^3^. This attrition can lead to decreased response quality, manifesting as an increase in measurement error^4^. Moreover, despite its improved sampling frequency, EMA might still yield a time series that is too sparse to accurately capture the underlying dynamics in moment-to-moment fluctuations in emotion and affect^4^. Therefore, the development of dense, passive, and unobtrusive smartphone measures that predict mental state is receiving increasing attention^5^.

Quantifying behavioural phenotypes using data collected unobtrusively from wearable digital devices is referred to as digital phenotyping^6^. One example of this approach is the open-science iOS app BiAffect^7^. Developed by our team, BiAffect replaces the user’s iPhone keyboard. It collects keyboard typing metadata (e.g., typing speed) as well as accelerometery data (movement and orientation while typing). Previous work has shown that typing speed predicts cognitive processing speed and shows an age-modulated, diurnal pattern^8,9^. In addition, several measures such as accelerometer displacement and autocorrect rate have been shown to predict depression or mania ratings^7,9,10^. These findings highlight the potential of passively collected typing data in clinical contexts.

While providing unique opportunities, the inception of this new technology requires analytical workflows that can extract meaningful behavioural phenotypes from the underlying timeseries. This poses several analytical challenges. Most importantly, it is often necessary to integrate data modalities that are acquired at different sampling frequencies^11^. This is important, for example, to validate the predictive power of digital phenotyping measures for mental health and cognition, most commonly against self-report measures^5^. However, self-report prompts tend to occur, at most, several times a day, which forms a data stream that is very sparse compared to the hundreds of daily samples collected by smartphones. Therefore, any study that aims to validate digital phenotyping measures of mental health must first address temporal misalignment. An additional problem arises when data are high-dimensional, making dimensionality reduction techniques desirable.

Some of these challenges have been recognised and addressed in the literature. For instance, deep, recurrent neural networks have been used to predict depression and mania scores from typing data, fusing different typing modalities (e.g., accelerometery and alphanumeric characters) either early or late in the network architecture^11,12^. Fusion to the much sparser depression and mania scores was achieved by stripping the temporal dimension from the typing modalities with gated recurrent units (GRUs). This contrasts with other approaches, where either the typing information is aggregated to the lower resolution of the emotional or cognitive scores^13^, or the scores are propagated (usually via interpolations) across all typing samples^14–16^.

The examples listed above all feature low-dimensional response variables. When considering high-dimensional responses instead, several dimensionality reduction techniques are available from the multivariate regression literature^17,18^. In the context of self-report data, previous research has utilised principal component analysis (PCA) to reduce high-dimensional self-report data to one component and study its dynamics over time^19^. Clustering approaches are an additional option for tackling high dimensionality in multivariate time series^20,21^.

The solution we employ involves: (1) applying temporal independent component analysis (ICA) to the high-dimensional modalities, (2) transforming all modalities to the same time unit through resampling or aggregation, and (3) then fusing the modalities through linear mixed-effects models as in prior work^15,22–25^. Temporal ICA decomposes a multivariate time series into a limited set of components by maximising their statistical independence in the time domain^23,24^. Crucially, ICA does not collapse the time domain, allowing classical resampling and aggregation techniques to align the generated independent components with the other digital phenotyping modalities. Additionally, ICA can compress data into a smaller number of independent components, making it ideally suited for dimensionality reduction. This means that fewer mixed-effects models need to be constructed, leading to a more parsimonious system of models that suffers less from multiple comparison corrections.

To demonstrate the value of our approach, we apply it to integrate high-quality self-report data with digital phenotyping data from the CLEAR-3 trial, a randomised controlled crossover trial that investigated how a hormonal intervention impacts menstrual cycle exacerbation of suicidal ideation and affective symptoms. The trial featured a unique clinical sample of mental health outpatients who were assigned female at birth (AFAB) and reported suicidal ideation in the past month. Participants self-reported on a large array of questionnaire items pertaining to affective, cognitive, and behavioural functioning on a daily basis and received substantial monetary compensation for the completion of daily ratings to ensure a high response rate that is not feasible in real-world applications. Meanwhile, they were encouraged to use the BiAffect iOS keyboard for the duration of the study.

We applied temporal ICA to the self-report data to distil the large number of items into fewer dimensions and predict their time course from BiAffect-derived data streams. Before running the ICA, we concatenated the self-report data of all participants along the temporal domain, both to increase the number of time steps fed into the analysis and to get a common set of independent components that applies to all participants^24,26^. Temporal ICA then takes this matrix of time series and decomposes it into a time-free mixing matrix and a set of components that are independent in the temporal domain. The mixing matrix specifies how the independent components combine to generate our original self-report measures.

**Fig. 1:**
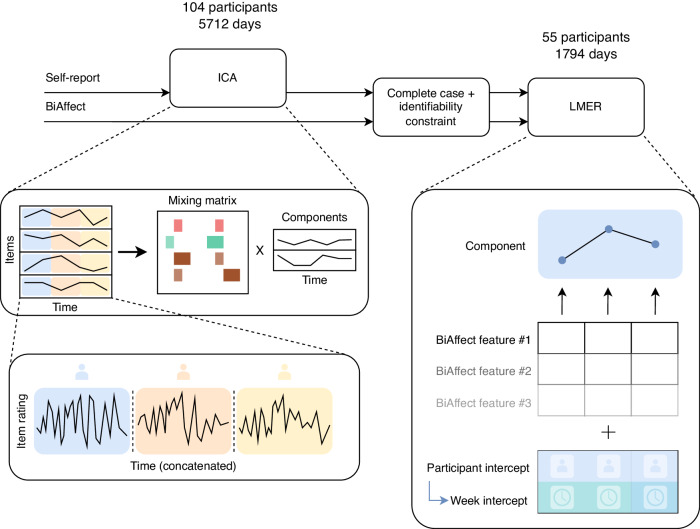
Overview of analysis pipeline. The ICA diagram displays how a collection of multiple time series (one series for every self-report item) gets decomposed into a mixing matrix (depicted as a bar diagram) and a reduced number of independent components with the time domain kept intact. Every item time series is constructed by concatenating the individual participant time series. The LMER diagram shows how the BiAffect features, along with participant- and week-within-participant-specific random intercepts, are used to predict the independent components. The complete case and identifiability constraints are specified in the Results. ICA independent component analysis, LMER linear mixed-effects regression.

Afterwards, we constructed a separate mixed-effects model for each component. These types of models allow us to separate our effects into fixed effects, which represent effects that theoretically apply to the entire population, and random effects, which represent individual departures from the fixed effects specific to the samples in our data^25^. More concretely, we added random intercepts per participant and per week within participant. All models were subjected to strict multiple comparison corrections. An overview of our approach is given in Fig. 1.

We demonstrate that our method yields a set of interpretable components of self-report data as well as stable associations between these components and keyboard-derived measures in a clinical sample with suicidal ideation.

## Results

### Demographics

**Fig. 2:**
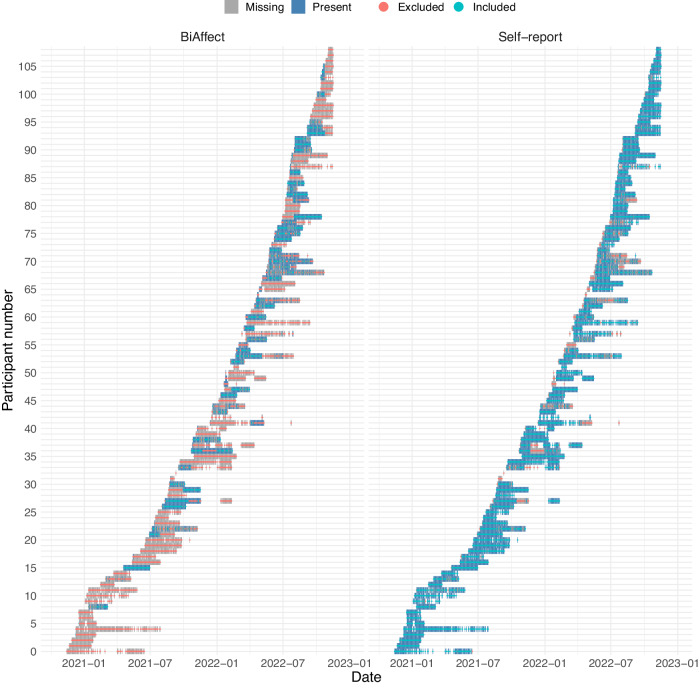
Missingness patterns of BiAffect and self-report data for all participants in the sample. Blank spaces (no grey or blue blocks) indicate that no records whatsoever were available for that date. For the self-report panel, all blocks that are present are also marked as included for the ICA analysis (cyan strikethrough), while those that do not are marked as excluded (red strikethrough). For the BiAffect panel, we included those data in the regression that were both present and fell within the included self-report range.

Our release of the CLEAR-3 data set contained 109 participants. Missing data patterns are given in Fig. 2. Some participants did not have self-report data in their baseline period, which meant that the ICA was run on 104 participants. Their demographics are given in Table 1. For the models, we included all BiAffect data that fell within the range of included self-report data (see Fig. 2).

**Table 1:** Participant demographics

	ICA	LMER
*N*	104	55
Age (mean (SD))	25.66 (4.63)	26.73 (4.87)
Race (%)		
Caucasian	49 (47.1)	26 (47.3)
African American	14 (13.5)	7 (12.7)
Asian	10 (9.6)	5 (9.1)
Don’t Know or More than one race	28 (26.9)	14 (25.5)
Unknown	3 (2.9)	3 (5.5)
Ethnicity (%)		
Hispanic	29 (27.9)	15 (27.3)
Non-Hispanic	73 (70.2)	38 (69.1)
Unknown	2 (1.9)	2 (3.6)
Education (%)		
High school degree, GED, or trade school	10 (9.6)	4 (7.3)
Post-graduate work	23 (22.1)	15 (27.3)
Some college or 2-year degree	28 (26.9)	13 (23.6)
4-year college degree	39 (37.5)	20 (36.4)
Unknown	4 (3.8)	3 (5.5)
Income (%)		
<$15,000	12 (11.5)	6 (10.9)
$15,000–$34,999	17 (16.3)	10 (18.2)
$35,000–$79,999	39 (37.5)	19 (34.5)
$80,000–$100,000	12 (11.5)	5 (9.1)
>$100,000	17 (16.3)	11 (20.0)
Unknown	7 (6.7)	4 (7.3)
Baseline clinical categories (%)		
Any current depressive disorder	64 (61.5)	32 (58.2)
Any current anxiety disorder	63 (60.6)	30 (54.5)
Any current obsessive-compulsive disorder	11 (10.6)	4 (7.3)
Any current substance use disorder	17 (16.3)	7 (12.7)
Any current eating disorder	12 (11.5)	6 (10.9)
Any current trauma-related disorder	25 (24.0)	9 (16.4)

*ICA* independent component analysis, *LMER* linear mixed-effects regression, *SD* standard deviation.

The LMER group is a subgroup of the ICA group.

Linear mixed-effects models require their cases to be complete, i.e., for one day, both BiAffect and self-report features needed to be present. We had a substantial number of incomplete days due to missing keyboard data, which led to the exclusion of 44 participants. These participants would sometimes choose or be forced to replace the BiAffect keyboard with their own keyboard due to, e.g., autocorrect and general usability issues (*n* = 4), multilingual requirements (*n* = 6), not having a suitable iOS device to install the app (*n* = 8), or technical issues (*n* = 4). Feedback from the remaining excluded participants was not available. We further required at least two observations for every week within a participant to allow identifiability of the random interaction between week and participant. We therefore excluded an additional 5 participants, leaving us with 55 participants. The demographics of this subgroup are given in Table 1. A grand total of 5712 days’ worth of data were fed into the ICA (average of 54.92 days per participant, *SD* = 28.54), while the mixed-effects models were built with 1794 days (average of 32.62 days per participant, *SD* = 19.95).

### Independent component analysis

Temporal ICA decomposes a matrix of time series into a time-free mixing matrix and a set of independent components. If the dimensionality of the independent components is smaller than the dimensionality of the original measures, the decomposition will also involve an error term (see Eq. (1)). In our case, the values of the mixing matrix indicate how much every estimated independent component contributes to the measured values of a self-report item (loading). We estimate the independent components by optimising for negentropy with the FastICA algorithm, as a higher negentropy implies less Gaussianity and thus more statistical independence^22^. In this work, we will interpret the ICA solutions by considering the mixing matrices, as they specify the link between the original self-report measures and the generated components.

**Fig. 3:**
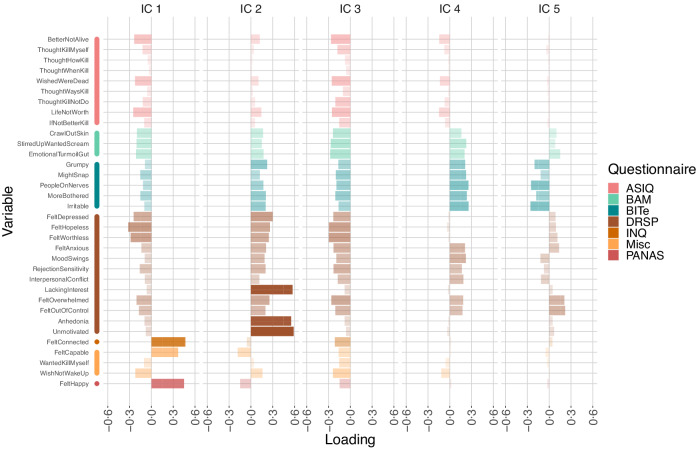
Mixing matrix of the 5-component decomposition of the self-report data. Bar opacity is an additional representation of the loading values. For questionnaire abbreviations, please refer to the main text. IC independent component.

The mixing matrix for a 5-component temporal ICA solution is shown in Fig. 3. (10- and 20-component solutions are shown in Supplementary Figs. 1 and 3 and compared to the 5-component solution in Supplementary Figs. 2 and 4). We selected 34 self-report items from the CLEAR-3 trial that pertained to various aspects of affective, cognitive, and behavioural functioning and were potentially relevant to acute suicidal ideation. These items are subsets of the Daily Record of Severity of Problems (DRSP)^27^, Brief Agitation Measure (BAM)^28^, Brief Irritability Test (BITe)^29^, Adult Suicidal Ideation Questionnaire (ASIQ)^30^, Positive and Negative Affect Schedule (PANAS)^31^, Interpersonal Needs Questionnaire (INQ)^32^, and some EMA items derived from a prior study (denoted here as ‘Miscellaneous’ or ‘Misc’). The exact questions corresponding to the self-report items are given in Supplementary Table 1. Since every column in Fig. 3 is linked to an independent component, we will refer to them by their component number.

IC 1 has large positive loadings for the FeltHappy, FeltCapable, and FeltConnected items, which are the only items in our set that pertain to positive affect. The loadings for the rest of the items are in the opposite, negative direction. We will therefore refer to this as the “well-being” component. This polarity pattern reappears for all investigated model orders, up to a reversal of the polarity (Supplementary Figs. 1, 3). IC 2 shows the same (reversed) pattern, but also displays large loadings for LackingInterest, Unmotivated, and Anhedonia, while the loadings for all other items are comparatively small. We will refer to this IC as the “anhedonia” component. IC 3 shows negative associations with all items in our set, possibly indicating a mean offset of which the intensity varies over time. E.g., if a participant gives consistently lower ratings than other participants, this might be represented with a higher IC 3 intensity. IC 4 gives positive loadings for items measuring agitation (BAM) and the related construct of irritability (BITe), as well as several DRSP items focused on interpersonal reactivity and conflict. We will refer to this IC as the “irritability and social dysfunction” component. Finally, IC 5 displays negative associations with the BITe and small, mixed loadings on the DRSP items. This mix makes it challenging to interpret this component, so we will refrain from naming it.

### Fusion with keyboard dynamics

The BiAffect preprocessing pipeline was based on previous studies^9,15^. In brief, all keyboard and accelerometery data were aggregated to the daily level. We extracted the following features: (1) median inter-key delay (IKD), an inverse measure of typing speed, (2) 95th percentile IKD, a measure of pausing within typing sessions, (3) mean absolute deviation (MAD) IKD, which quantifies typing speed variability^9^, (4) autocorrect rate, (5) backspace rate, (6) the total number of key presses per day, (7) the percentage of typing sessions spent upright, and (8) the percentage of typing sessions where the phone recorded movement.

Our mixed-effects models contained fixed effects for all BiAffect features, random intercepts for participants, and random interactions between week and participant. We found no gross violations of model assumptions. For the remainder of this section, we have declared any effects with (corrected) *p* values beneath α = 0.05 significant. Forwards-fitting of the random effects indicated that the interaction of week and participant was a significant addition to all models (for all models, *p* < 0.0001). Model parameter estimates are given in Table 2 (for the estimates of the 10- and 20-component solutions, see Supplementary Tables 2, 3). To account for the problem of multiple comparisons, we applied stringent Bonferroni corrections to the associated *p* values. This stringency means we increase our risk of type II errors, so we also provide uncorrected *p* values for transparency. After Bonferroni correction, we found that less phone movement corresponded to more anhedonia (IC 2) on the same day (β = −0.12, 95% CI [−0.17, −0.07], *p* = 0.00030). As for terms with *p* < 0.05 only in the uncorrected case, we found that increased movement rate was associated with greater well-being (β = 0.071, 95% CI [0.02, 0.12], uncorrected *p* = 0.0051) in the IC 1 model, higher median IKD (slower typing) predicted more anhedonia (β = 0.098, 95% CI [0.02, 0.18], uncorrected p = 0.013) in the IC 2 model, lower median IKD (faster typing; β = −0.094, 95% CI [−0.18, −0.01], uncorrected *p* = 0.030) and a higher total number of key presses (β = 0.062, 95% CI [0.01, 0.12], uncorrected *p* = 0.027) predicted more irritability and social dysfunction in the IC 4 model, and a lower total number of key presses predicted higher IC 5 intensity (β = −0.060, 95% CI [−0.11, −0.01], uncorrected *p* = 0.025).

**Table 2:** Mixed-effects model estimates with their uncorrected and corrected *p* values

	IC 1			IC 2			IC 3			IC 4			IC 5		
	β	p'	p	β	p'	p	β	p'	p	β	p'	p	β	p'	p
Median IKD	0.034	0.36	1	0.098	0.013	0.52	0.023	0.52	1	−0.094	0.030	1	0.018	0.66	1
95th percentile IKD	−0.013	0.68	1	−0.030	0.36	1	−0.015	0.63	1	0.057	0.12	1	−0.023	0.51	1
MAD IKD	−0.0022	0.94	1	−0.027	0.42	1	0.043	0.16	1	0.053	0.14	1	0.0016	0.96	1
Autocorrect rate	0.015	0.58	1	−0.00029	0.99	1	−0.034	0.20	1	0.022	0.47	1	−0.015	0.62	1
Backspace rate	−0.016	0.51	1	0.032	0.20	1	0.018	0.45	1	−0.0045	0.87	1	0.0047	0.86	1
Total number of key presses	−0.00083	0.97	1	0.022	0.39	1	−0.030	0.21	1	0.062	0.027	1	−0.060	0.025	1
Movement rate	0.071	0.0051	0.20	−0.12	<0.0001	0.00030	1.4e-05	1	1	0.055	0.058	1	−0.020	0.48	1
Upright rate	0.037	0.14	1	0.016	0.55	1	0.026	0.29	1	0.017	0.56	1	−0.027	0.32	1

*IC* independent component, *IKD* inter-key delay, *MAD* mean absolute deviation.

Every IC corresponds to a separate model. p’ indicates uncorrected *p* values, *p* indicates Bonferroni-corrected *p* values.

### Supplementary analyses

We investigated the stability of the ICA solutions across multiple FastICA restarts and found that in most cases the well-being and anhedonia components would combine into one component that indicated general affect (Supplementary Fig. 5). Phone movement remained a significant predictor of anhedonia. When the ICA solutions featured the general affect component instead, we found that phone movement also significantly predicted the general component.

A forwards-fitting procedure was run for the random effects to examine the influence of random slopes on the fixed-effect estimates (Supplementary Table 5). The addition of random slopes did not change any of the conclusions drawn from the base models described above.

Some of our data included periods with high proportions of missing data. We repeated our random restart analysis with only contiguous subsets of the data to assess the influence of missingness on our results (Supplementary Fig. 6). In some cases, phone movement did no longer significantly predict anhedonia after multiple comparison corrections. Potential reasons for this behaviour are given in the supplement.

To verify that IC 3 represented a mean offset of self-report responses for certain participants, we correlated the average responses with the IC 3 values and found a negative correlation (Supplementary Fig. 7). In addition, we reran our analysis after within-participant mean-centring of the self-report data and found that in this case IC 3 disappeared (Supplementary Fig. 8). The implications of this finding are discussed in the supplement (see also Supplementary Table 4).

## Discussion

In this work, we introduce a generic method for the analysis and integration of digital phenotyping with self-report data. It utilises temporal ICA to extract interpretable components from the data while keeping the temporal dimension of the data intact, providing a principled method to align different data modalities. We validated the method in a dataset acquired from participants with a history of suicidal ideation and found well-being, anhedonia, and irritability and social dysfunction components in the high-dimensional self-report data. This low-dimensional representation could be predicted by smartphone typing dynamics and accelerometery in that more phone movement while typing was associated with less anhedonia. We demonstrate that passively collected smartphone keyboard dynamics are predictive of a low-arousal state in people with suicidal ideation, as measured using extensive validated instruments that are hard to deploy at scale.

Our method aligned self-report and keyboard dynamics data, allowing their joint analysis and providing further evidence for the use of keyboard dynamics as an ecologically derived marker of mental well-being. Moreover, we distilled high-dimensional data into interpretable components that can be related to the existing literature more easily.

For instance, the emergence of the wellbeing, anhedonia, and irritability and social dysfunction components, as identified by our temporal ICA, can be interpreted in the light of the core affect framework^33^. According to the core affect theory, core affect is “a neurophysiological state that is consciously accessible as a simple, non-reflective feeling” and is a blend of two dimensions: Pleasure-displeasure and activation-deactivation^33^. Our components are easily mapped onto this domain. The well-being component aligns mainly with the pleasure dimension and is quite neutral w.r.t. activation. Anhedonia, on the other hand, indicates low activation and a small amount of displeasure. The irritability and social dysfunction component most likely is a blend of high activation and displeasure. Since core affect is postulated to be involved in emotional episodes^33^, it is encouraging that our temporal ICA is able to identify components that can readily be compared to the core affect dimensions and predicted with data collected passively using smartphones.

Specifically, we showed that phone movement was predictive of the anhedonia component, which is intuitively understandable in that less movement while typing is associated with more anhedonia, a higher lack of interest and a higher lack of motivation. Additionally, we found that phone movement was a trend significant, positive predictor of well-being, which complements the relationship between movement and anhedonia. We only found a small set of prior literature to compare these findings to. Zulueta et al., for example, reported that more phone movement (calculated differently than in our study) predicted higher depression and mania ratings in a sample of participants with bipolar disorder^7^. However, they also pointed out that bipolar depression can manifest as either psychomotor retardation or agitation^34^, and therefore their results may not be directly comparable to our findings.

Considering the relationship between movement and anhedonia more generally, it appears that this relationship has not been fully explored as evidenced by the scarce literature we could identify. However, Sierra et al. pointed towards the denervation of dopaminergic D2/D3 receptors as being a root cause of apathy (lack of motivation) in Parkinson’s disease^35^; in the work of Treadway and Zald, this apathy is referred to as motivational anhedonia^36^. Lemke et al. have investigated the relationship between anhedonia and psychomotor retardation in depression and found them to be correlated^37^, which is consistent with our findings, but their work was published in 1999, when passive sensing was not feasible. Given that we have not found other studies that utilise passive sensing in this context, our findings thus support the role of digital phenotyping as a valuable tool for investigating this relationship more closely.

In addition, we found the trend significant effect that larger IKDs (slower typing) predict more anhedonia and that smaller IKDs (faster typing) are related to increased levels of irritability and social dysfunction. While we refrain from drawing conclusions due to non-significance after (stringent) multiple comparison correction and a lack of prior literature, it is notable that our findings conform to intuition. Our data also suggest that more key presses predict higher levels of irritability (IC 4 and 5). We note that previous digital phenotyping studies have used measures related to data quantity (e.g., the duration of periods of successful data collection) to detect schizophrenia severity and relapses^38,39^, which suggests the utility of employing typing dynamics quantity metrics for similar purposes. This would be an interesting direction for future work.

Our work is embedded in a broader effort to fuse typing dynamics with measures of affect and mental well-being. As noted in the introduction, previous work has used classification models to predict depression and mania scores from typing data with high accuracy^11,13,14^. Our approach, on the other hand, focuses on regression rather than classification, which avoids the necessity to discretise a continuous outcome measure. There are additional studies that also used regression to link typing dynamics to other modalities^7–10,12,15,16^, but they do not consider the case of high-dimensional response variables. In addition, many of these studies have been conducted with relatively small sample sizes (*n* ≤ 26)^8,10–12,14,16^, whereas our sample is moderately sized and deeply phenotyped.

While we have demonstrated our approach within the context of typing dynamics, other studies have collected a wide variety of other digital phenotyping modalities, such as social behaviour (e.g., outgoing phone calls)^40,41^, GPS patterns^40–42^, and actigraphy^41,43,44^. (For a more complete overview of possible modalities, see the work of Melcher, Hays, and Torous^45^) Many of these studies also involve the collection of EMA data^45^, presenting them with similar problems of temporal misalignment as well as high dimensionality. Because these problems are not fundamentally different from what we encountered with typing data, we believe that our method will be equally beneficial for the integration of these alternative data types.

There are several caveats and limitations that we would like to highlight. First, the more components we request from the ICA, the more challenging their interpretation becomes. Many components of the 10- and 20-component solutions, for instance, contain a mix of positive and negative loadings for items from a single scale, which would suggest that such components represent either very specific aspects of a domain or just capture noise (Supplementary Figs. 1 and 3). On the other hand, we stress that low-dimensional ICA solutions need not be the optimal ones. Other decompositions might be equally valid, depending on the level of granularity one wishes to examine^46^.

In a similar vein, the run-to-run variability of the solutions from the FastICA algorithm poses an additional challenge to their interpretability and subsequent clinical utility. Our data lend themselves to multiple low-dimensional representations and it might be unclear which representation is the most adequate. Nevertheless, we also view the multiplicity of the ICA solutions as one of the strengths of our method, as they can be compared to explore the possible groupings of the self-report items rather than forcing the ICA solutions to conform to just one of the possible low-dimensional representations. We have therefore performed multiple random restarts to assess the stability of these representations, an approach that is common with machine learning algorithms that exhibit stochasticity in their solutions (see, for an example specific to ICA, the well-validated ICASSO tool^47^). A similar approach could be used for making robust predictions. As Supplementary Fig. 5 shows, the solutions fall in a small number of distinct and consistent categories for our data. If the number of distinct clusters is reasonable, interpretation need not be difficult.

Second, Fig. 2 showed that in some participants substantial portions of the BiAffect data are missing. Indeed, some participants strongly preferred the autocorrect behaviour of the native iOS keyboard, whilst others had multilingual requirements that were not supported by the current version of the English-only BiAffect keyboard or were experiencing technical issues. We did not receive feedback from all participants that stopped using the keyboard, so there could be other factors contributing to the observed attrition, but we believe that these aspects could be the focus of future improvement. The aforementioned limitations mainly exist because we developed BiAffect and its autocorrect functionalities in-house; they should not be inherent to keyboard typing dynamics itself. We also point out that high proportions of missing data are prevalent in most digital phenotyping studies. With ICA as the core part of our processing pipeline, we can handle this missingness under stationarity conditions. Moreover, the fact that, due to our study incentivisation, the proportions of missingness for data that require active participation (self-report) are much lower than those of passively collected data (BiAffect) is an exception rather than the norm compared to other studies^39^.

Third, while we have found that phone movement while typing is predictive of anhedonia, it is not entirely clear if and how our anhedonia component contributes to suicidal ideation. Links have been found between arousal and suicidal behaviour^48^, but more research is needed to determine how our anhedonia component maps onto the arousal operationalisations used in the literature. Once this mapping has been clearly delineated, we can potentially leverage the fluctuations in phone movement as part of an early warning system for heightened levels of suicidal ideation.

Finally, the digital phenotyping analysis toolbox is still far from complete. For instance, not much is known about the autocorrelation properties of keyboard and accelerometery dynamics. While our previous research has identified diurnal patterns in keyboard dynamics^9^, it is not unlikely that there are weekly, monthly, or even seasonal patterns in the BiAffect features, warranting further research.

To conclude, temporal ICA is an effective tool to decompose high-dimensional, daily self-report data without collapsing the time domain. In our dataset containing affective self-report data of people assigned female sex at birth with a history of suicidal ideation, we found ICA-based representations of affect that mapped onto digital phenotyping measures in an interpretable fashion, namely as wellbeing, anhedonia, and irritability and social dysfunction components consistent with the pleasure and activation axes found in core affect theory. We consider this method to be widely applicable and a valuable contribution to the methods toolbox for analysing densely sampled longitudinal and digital phenotyping data.

## Methods

### Study design

Our study utilised data from the CLEAR-3 trial, a randomised controlled crossover trial that investigated how perimenstrual administration of estradiol (E) and progesterone (P), relative to natural steroid withdrawal under placebo, impacts menstrual cycle exacerbation of suicidal ideation and affective symptoms (NCT04112368). The study was approved by the UIC Institutional Review Board and all relevant ethical regulations were adhered to. Data acquisition for this trial was ongoing, so only baseline (pre-experimental) data were used for the present study, which consisted of at least a full menstrual cycle.

### Recruitment and exclusion criteria

All participants were assigned female sex at birth (AFAB), reported past-month suicidal ideation (SI), and were in outpatient treatment. Participants, who were recruited from the community via social media ads and received up to US$1250 after completing the entire trial, were 18–45 years of age, had normal menstrual cycles (25–35 days), did not take any hormonal medications, and had normal weight (BMI 18–29). Exclusion criteria included any long-term nonpsychiatric health condition, a history of hospitalisation for mania or psychosis, or any affective or substance use disorder deemed likely to interfere with safe participation in the clinical trial. All participants provided informed consent for study participation.

### Keypress data preprocessing

The keypress data were aggregated in two steps. First, individual keypresses were aggregated into typing sessions, which begin as soon as the user presses the first key and end when the keyboard is no longer displayed or after six seconds of inactivity^15^. For each session, the number of autocorrect and backspace presses were counted and divided by the total number of keypresses in the session to get the autocorrect and backspace rates. In addition, the total number of keypresses was counted. Finally, the inter-key delays (IKDs) were calculated between all successive alphanumeric keypresses in the session. From these IKDs, we calculated (1) the median IKD, an inverse measure of typing speed, (2) the 95th percentile IKD, a measure of pausing within sessions, and (3) the mean absolute deviation (MAD) IKD, which quantifies typing speed variability^9^.

After session-level aggregation, the sessions were aggregated to the daily level by taking the mean of all session-level variables. The one exception to this rule were the total numbers of session key presses, which were simply summed across the day. Any days with less than 750 key presses were excluded from further analysis to ensure proper feature estimation.

Finally, the number of key presses was log-transformed and all BiAffect features were standardised w.r.t. the entire sample (i.e., grand mean set to 0 and overall variance to 1) to aid model fitting.

### Accelerometer data preprocessing

Our accelerometer data only included samples collected while the participant was typing on the BiAffect keyboard. While collecting data all throughout the day would yield more data, it would also be more taxing for the smartphone battery, and offloading the accelerometer recording to an external device would require participants to wear an extra device.

Accelerometer data were grouped into typing sessions and low-pass filtered using a second-order bidirectional Butterworth filter with a cutoff frequency of 4 Hz to remove noise^15^. Afterwards, every sample within a session was classified as either moving (also ‘active’) or stationary based on the magnitude of the filtered x, y, and z accelerometer readings. Magnitudes close to 1 reflect the natural gravitational pull of the earth and therefore indicate the user’s phone is at rest. We classified samples with a magnitude below 0.95 and above 1.05 as active, and everything that fell within these (inclusive) bounds as stationary. An entire session was classified as active if over 8% of its constituent samples was classified as active.

In addition, each session was classified as upright or not using the median values of the filtered x and z values. If the median z value of a session was (strictly) below 0.1 and the median x value was in-between −0.2 and 0.2 (inclusive), the session was classified as upright^15^. Sessions that were not classified as upright could potentially indicate that participants were using their phone while lying down.

Finally, by counting the number of active and upright sessions within a day and dividing those counts by the total number of sessions within a day, we get a rate of active and a rate of upright sessions per day.

### Independent component analysis

In general, an ICA will decompose a data matrix $X$ into a mixing matrix $A$ and a source matrix $S$ such that^22^:

1 $$X={AS}+\epsilon ,$$

where *ϵ* represents an error term. *X* is a $p$ by $n$ matrix, where $p$ indicates the number of self-report items and $n$ corresponds to the number of days of data. In our case, we concatenate the data of all participants along the time axis to (1) ensure $n$ is sufficiently large to run the ICA and (2) receive a single set of independent components that applies to all participants. $A$ is a $p$ by $q$ and $S$ is a $q$ by $n$ matrix, where $q$ indicates the number of components we would like the data to be reduced to. Note that if $q=p$, $\epsilon =0$. In other words, if we request as many components as there are self-report items, there is no error and $X$ is exactly equivalent to ${AS}$.

We used the FastICA algorithm to estimate $A$ and $S$ from the original self-report data^22^. More specifically, we used the parallel version with $G$ set to the log cosh function and ${a}_{1}=1$, implemented in *R* (version 4.2.2) by the *fastICA* package (version 1.2.3).

ICAs are typically run on continuous data that can take negative values, while our self-report data consisted of strictly positive Likert scales. We therefore log-transformed all self-report measures prior to running the ICA.

Solving for independent components typically necessitates a stochastic optimisation and therefore has associated run-to-run variability. We ran a sensitivity analysis to check the extent of this variability (Supplementary Fig. 5).

### Data fusion models

We used mixed-effects models to fuse BiAffect data to the self-report components. Mixed-effects models divide their effects into fixed effects, which are considered the effects that (in theory) apply to the entire population, and random effects, which represent deviations from the fixed effects that are due to the specifics of our sample^25^. A typical example of random effects is the participant-specific deviation from the global mean in a repeated-measures experiment. In our study, the self-report data did indeed show such participant-specific deviations. Moreover, we found evidence that, depending on the participant, there were week-to-week deviations as well. Such deviations are not unsurprising, as a specific week might have been good for some participants, while others might have experienced it as a particularly bad one. The menstrual cycle of our participants is also likely to bring about periodic fluctuations in the self-report data that can be captured on the weekly level. We therefore opted for modelling a random effect of week nested within participants. In other words, we estimate a random intercept per participant, as well as a random interaction between week and participant.

Formulating such a model for ${N}_{{part}}$ participants with a participant-dependent ${N}_{{week}}$ number of weeks gives:

2 $${y}_{{ijk}}={\beta }_{0}+{b}_{i}+{b}_{{ij}}+{{\boldsymbol{\beta }}}_{{\bf{1}}}^{{{\top }}}{{\boldsymbol{x}}}_{{\boldsymbol{ijk}}}{\boldsymbol{+}}{\epsilon }_{{ijk}},$$

where $i=1,\ldots ,{N}_{{part}}$ is the participant index, $j=1,\ldots ,{N}_{{week}}$ is the week index, and $k=1,\ldots ,7$ is the day-of-week index. ${y}_{{ijk}}$ represents an independent component (IC) value for participant $i$ in week $j$ on day $k$ and is our dependent variable. ${\beta }_{0}$ is the grand mean of the IC values across all participants and weeks. ${b}_{i}$ denotes the random effect of participant, i.e., how much participant $i$ shifts the grand mean, on average. Similarly, ${b}_{{ij}}$ indicates how much week $j$ shifts the participant-specific mean ${\beta }_{0}+{b}_{i}$, but only for participant $i$. ${{\boldsymbol{x}}}_{{\boldsymbol{ijk}}}$ is the BiAffect feature (column) vector, which holds a value for the inter-key delay, autocorrect rate, phone movement rate, et cetera. Like ${y}_{{ijk}}$, it is specific to participant $i$ in week $j$ on day $k$. In contrast, the parameter vector ${{\boldsymbol{\beta }}}_{{\boldsymbol{1}}}^{{\boldsymbol{\top }}}$ does not depend on a specific participant, week, or day: This vector represents our fixed effects. (The superscripted $\top$ denotes the transpose, converting the column vector into a row vector.) Finally, the model has an error term ${\epsilon }_{{ijk}}$, which incorporates all IC variation that is not captured by the rest of our model.

We assume that all random effects and the error term are normally distributed around 0. In other words:

3 $${b}_{i}{\mathscr{ \sim }}{\mathscr{N}}\left(0,{\sigma }_{1}^{2}\right),{b}_{{ij}}{\mathscr{ \sim }}{\mathscr{N}}\left(0,{\sigma }_{2}^{2}\right),{\epsilon }_{{ijk}}{\mathscr{ \sim }}{\mathscr{N}}\left(0,{\sigma }^{2}\right),$$

where ${\sigma }_{1}^{2}$, ${\sigma }_{2}^{2}$, and ${\sigma }^{2}$ represent the variance of, respectively, the participant random intercept, the random interaction between participant and week, and the error.

We used the *nlme* package (version 3.1.160) for *R* to create these models. We determined conformity to the linear mixed-effects model assumptions by visual assessment. To assess normality of the residuals and random effects, we examined their QQ plots. Heteroskedasticity was judged by plotting the standardised residuals versus the fitted values. We constructed a separate model for each IC of an ICA solution and employed Bonferroni corrections across all models for that solution to avoid multiple comparison problems. In particular, the Bonferroni correction factor ${CF}$ was defined as:

4 $${CF}={n}_{{IC}}\cdot {n}_{{fixed}},$$

where ${n}_{{IC}}$ indicates the number of ICs and ${n}_{{fixed}}=8$ represents the number of fixed-effect terms (excluding the intercept).

### Reporting summary

Further information on research design is available in the Nature Research Reporting Summary linked to this article.

### Supplementary information

Supplement

Reporting Summary

## Data Availability

Deidentified participant data will be made available on reasonable request to the principal investigator of the CLEAR-3 trial, T.A. Eisenlohr-Moul (temo@uic.edu).

## Code availability

All analysis source code is freely available on GitHub: https://github.com/Valkje/clear3-ica.
