# Supplementary material for: Smartphone keyboard dynamics predict affect in suicidal ideation
Source: NPJ Digit Med. 2024 Mar 1;7:54. doi: 10.1038/s41746-024-01048-1 (PMC10907683; doi:10.1038/s41746-024-01048-1)
Supplement: Supplementary file 2 — Reporting Summary [file 41746_2024_1048_MOESM2_ESM.pdf]

Corresponding author(s): Loran Knol

Last updated by author(s): Dec 12, 2023

Reporting Summary

Nature Portfolio wishes to improve the reproducibility of the work that we publish. This form provides structure for consistency and transparency in reporting. For further information on Nature Portfolio policies, see our Editorial Policies and the Editorial Policy Checklist.

Statistics

For all statistical analyses, confirm that the following items are present in the figure legend, table legend, main text, or Methods section.

n/a	Confirmed	
☐	☒	The exact sample size (*n*) for each experimental group/condition, given as a discrete number and unit of measurement
☐	☒	A statement on whether measurements were taken from distinct samples or whether the same sample was measured repeatedly
☐	☒	The statistical test(s) used AND whether they are one- or two-sided<br>*Only common tests should be described solely by name; describe more complex techniques in the Methods section.*
☐	☒	A description of all covariates tested
☐	☒	A description of any assumptions or corrections, such as tests of normality and adjustment for multiple comparisons
☐	☒	A full description of the statistical parameters including central tendency (e.g. means) or other basic estimates (e.g. regression coefficient) AND variation (e.g. standard deviation) or associated estimates of uncertainty (e.g. confidence intervals)
☐	☒	For null hypothesis testing, the test statistic (e.g. *F*, *t*, *r*) with confidence intervals, effect sizes, degrees of freedom and *P* value noted<br>*Give P values as exact values whenever suitable.*
☒	☐	For Bayesian analysis, information on the choice of priors and Markov chain Monte Carlo settings
☒	☐	For hierarchical and complex designs, identification of the appropriate level for tests and full reporting of outcomes
☐	☒	Estimates of effect sizes (e.g. Cohen's *d*, Pearson's *r*), indicating how they were calculated

Our web collection on statistics for biologists contains articles on many of the points above.

Software and code

Policy information about availability of computer code

Data collection	Self-report and typing data was collected through the BiAffect iOS app.
Data analysis	For our analyses, we used R (version 4.2.2) and its fastICA (version 1.2.3) and nlme (version 3.1.160) packages. All our custom code is publicly available on GitHub: https://github.com/Valkje/clear3-ica

For manuscripts utilizing custom algorithms or software that are central to the research but not yet described in published literature, software must be made available to editors and reviewers. We strongly encourage code deposition in a community repository (e.g. GitHub). See the Nature Portfolio guidelines for submitting code & software for further information.

Data

Policy information about availability of data

All manuscripts must include a data availability statement. This statement should provide the following information, where applicable:

- Accession codes, unique identifiers, or web links for publicly available datasets
- A description of any restrictions on data availability
- For clinical datasets or third party data, please ensure that the statement adheres to our policy

Deidentified participant data will be made available on reasonable request to the principal investigator of the CLEAR-3 trial, T.A. Eisenlohr-Moul (temo@uic.edu), when provided with an appropriate analysis plan.

Research involving human participants, their data, or biological material

Policy information about studies with human participants or human data. See also policy information about sex, gender (identity/presentation), and sexual orientation and race, ethnicity and racism.

Reporting on sex and gender	Since the CLEAR-3 trial investigates the menstrual cycle, all of our participants were assigned female sex at birth. We did not disaggregate our data by gender.
Reporting on race, ethnicity, or other socially relevant groupings	We did not analyse our data by race, ethnicity, or any other socially relevant grouping.
Population characteristics	See below.
Recruitment	Participants were recruited from the community via social media ads and received up to US$1,250 after completing the entire trial. The social media ads were targeted to those specifying female sex and age 18-44 on their Instagram or Facebook profile; otherwise, they were open.
Ethics oversight	UIC Institutional Review Board

Note that full information on the approval of the study protocol must also be provided in the manuscript.

Field-specific reporting

Please select the one below that is the best fit for your research. If you are not sure, read the appropriate sections before making your selection.

☐ Life sciences ☒ Behavioural & social sciences ☐ Ecological, evolutionary & environmental sciences

For a reference copy of the document with all sections, see nature.com/documents/nr-reporting-summary-flat.pdf

Behavioural & social sciences study design

All studies must disclose on these points even when the disclosure is negative.

Study description	Quantitative longitudinal study
Research sample	Since the CLEAR-3 trial investigates the effect of a hormonal intervention on suicidal ideation surrounding menses, our sample contained people assigned female sex at birth (AFAB), with a mean age of 25.66 (SD = 4.63). Participants were recruited from the local Chicago community on social media. Our sample is representative insofar as the population of AFAB people that experience perimenstrual suicidal ideation are well-represented on social media.
Sampling strategy	Convenience sampling. With 85 participants that completed the entire CLEAR-3 trial, the trial would have 80% power to detect small-to-medium sized effects (f-squared = .10). Effects sized smaller than that would not be clinically meaningful.
Data collection	For the purposes of the present study, we collected data using the BiAffect iOS app, which both measured keyboard and accelerometery dynamics and delivered the self-report assessments. This happened in participants' daily lives, all throughout the day.
Timing	The first sample was collected on 13 November 2020. The last sample included in our study was from 16 November 2022.
Data exclusions	We excluded 5 participants due to an absence of self-report data, 44 participants due to absent keyboard data, and an additional 5 participants due to insufficient levels of merged self-report and keyboard data for correct identification of week-within-subject-level random intercepts. So, in total, we excluded 54 participants.
Non-participation	During the course of the study, 16 participants dropped out of our initial sample. Reasons for drop-out were (medical) ineligibility, participants not having enough time for participation, and loss to follow-up.
Randomization	Randomisation was performed after the baseline period in the CLEAR-3 trial. Note that the present study only used baseline data, but did not make any group comparisons.

Reporting for specific materials, systems and methods

We require information from authors about some types of materials, experimental systems and methods used in many studies. Here, indicate whether each material, system or method listed is relevant to your study. If you are not sure if a list item applies to your research, read the appropriate section before selecting a response.

Materials & experimental systems

n/a	Involved in the study
☒	☐ Antibodies
☒	☐ Eukaryotic cell lines
☒	☐ Palaeontology and archaeology
☒	☐ Animals and other organisms
☐	☒ Clinical data
☒	☐ Dual use research of concern
☒	☐ Plants

Methods

n/a	Involved in the study
☒	☐ ChIP-seq
☒	☐ Flow cytometry
☒	☐ MRI-based neuroimaging

Clinical data

Policy information about clinical studies

All manuscripts should comply with the ICMJE guidelines for publication of clinical research and a completed CONSORT checklist must be included with all submissions.

Clinical trial registration

NCT04112368

Study protocol

The protocol can be retrieved from https://www.clinicaltrials.gov/study/NCT04112368

Data collection

Data were collected in Chicago, IL, USA, as well as from the participants' own smartphones. Recruitment started on 2020-09-15 and is ongoing.

Outcomes

For the purposes of the current study, which only assessed baseline data, the primary and secondary outcomes of the CLEAR-3 trial were not assessed.

Plants

Seed stocks

Report on the source of all seed stocks or other plant material used. If applicable, state the seed stock centre and catalogue number. If plant specimens were collected from the field, describe the collection location, date and sampling procedures.

Novel plant genotypes

Describe the methods by which all novel plant genotypes were produced. This includes those generated by transgenic approaches, gene editing, chemical/radiation-based mutagenesis and hybridization. For transgenic lines, describe the transformation method, the number of independent lines analyzed and the generation upon which experiments were performed. For gene-edited lines, describe the editor used, the endogenous sequence targeted for editing, the targeting guide RNA sequence (if applicable) and how the editor was applied.

Authentication

Describe any authentication procedures for each seed stock used or novel genotype generated. Describe any experiments used to assess the effect of a mutation and, where applicable, how potential secondary effects (e.g. second site T-DNA insertions, mosiacism, off-target gene editing) were examined.
